# Surgical Ulcer Debridement in a Patient With Spina Bifida Complicated by Osteomyelitis, Obesity, and Diabetes

**DOI:** 10.7759/cureus.43470

**Published:** 2023-08-14

**Authors:** Gabriella L Bruzzese, Stephen McKenna

**Affiliations:** 1 Department of General Surgery, Frederick Health Hospital, Frederick, USA; 2 Department of Anatomy, West Virginia School of Osteopathic Medicine, Lewisburg, USA

**Keywords:** hydrosurgery, chronic pain, pressure injury, neuropathic ulceration, buttock, bariatric medicine, debridement and lavage, sacral pressure ulcer, ostemyelitis, spina bifida

## Abstract

The combination of obesity, diabetes mellitus (DM), and reduced mobility due to spina bifida can contribute to an increased risk of osteomyelitis. Spina bifida, a congenital defect of the spinal cord, causes vertebral column deformities and neurological impairment. Obesity can lead to increased pressure and stress on the bones and joints, as well as poor circulation and immune dysfunction, including neutrophil migration disorders. Similarly, DM can also contribute to poor circulation and inadequate immune function. These changes can increase the risk of neuropathic ulcerations and osteomyelitis. We report a case of a 59-year-old man who presented for surgical consultation at the inpatient care unit. He had a nonhealing sacral ulcer on the left buttock that persisted for a year. He had a history of spina bifida, type 2 DM, and anemia, and his body mass index was 57.6 kg/m^2^. Physical examination revealed an unstageable left buttock pressure ulcer. The patient was afebrile, and his laboratory findings and imaging results indicated osteomyelitis. Despite intravenous antibiotic treatment, healing of the sacral ulcer remained poor, and the patient experienced chronic pain. Subsequent surgical intervention in the operating room involved debridement of the skin and soft tissue using high-powered water via the VERSAJET™ Hydrosurgery System (Smith & Nephew, London, UK). Ulcerated and necrotic skin and subcutaneous tissue extending deeper than the muscles and bones were observed. Loop colostomy was performed after determining that the wound would not heal owing to its proximity to the rectum and the inevitable contamination with stool. Postoperatively, sacral bone biopsy confirmed osteomyelitis. Alternative treatment options remained limited, as several other treatment methods failed prior to surgical debridement and colostomy placement. Although repeated debridement improved tissue viability, loop colostomy was performed to divert stool and prevent contamination of the ulcer.

## Introduction

Obesity, diabetes mellitus (DM), and spina bifida can individually and synergistically contribute to an increased risk of osteomyelitis. Spina bifida is a congenital spinal cord defect that can lead to physical deformities of the vertebral column or neurological defects or both. Patients diagnosed with spina bifida are at an increased risk of osteomyelitis for several reasons, such as paralysis or weakness in the lower limbs, resulting in decreased sensation and mobility [1]. This can lead to skin breakdown or pressure injuries, facilitating the entry of bacteria into the body. Furthermore, individuals with spina bifida might also exhibit structural abnormalities in their bones and joints, exacerbating susceptibility to bacterial infections. Therefore, these individuals are at an increased risk of developing chronic wounds [2]. A compromised immune system, commonly observed in patients with spina bifida, can further increase the risk of infections, including osteomyelitis [3-5]. The risk is further compounded when spina bifida is complicated by obesity and DM. Obesity can lead to increased pressure and stress on the bones and joints, and the associated excessive mechanical load can cause microtrauma and damage to the bone’s protective barrier, making it more susceptible to infections, such as osteomyelitis [6]. DM is characterized by chronically elevated blood sugar levels, which can damage blood vessels and impair blood flow to various parts of the body, including the bones. Poor circulation in the affected area can compromise the delivery of oxygen and nutrients to the bone, hindering its ability to heal and making it more vulnerable to infections, such as osteomyelitis [7]. Moreover, both obesity and DM are associated with impaired immune function [8,9]. When these three conditions coexist in an individual, the risk of osteomyelitis is increased due to the combined effects of compromised bone integrity, impaired immune function, and reduced sensation or awareness of potential injuries [1,3,5]. This study reports a case of a patient with spina bifida, demonstrating the rarity of sacral osteomyelitis and highlighting the complexity of the patient’s condition.

## Case presentation

**Figure 1:**
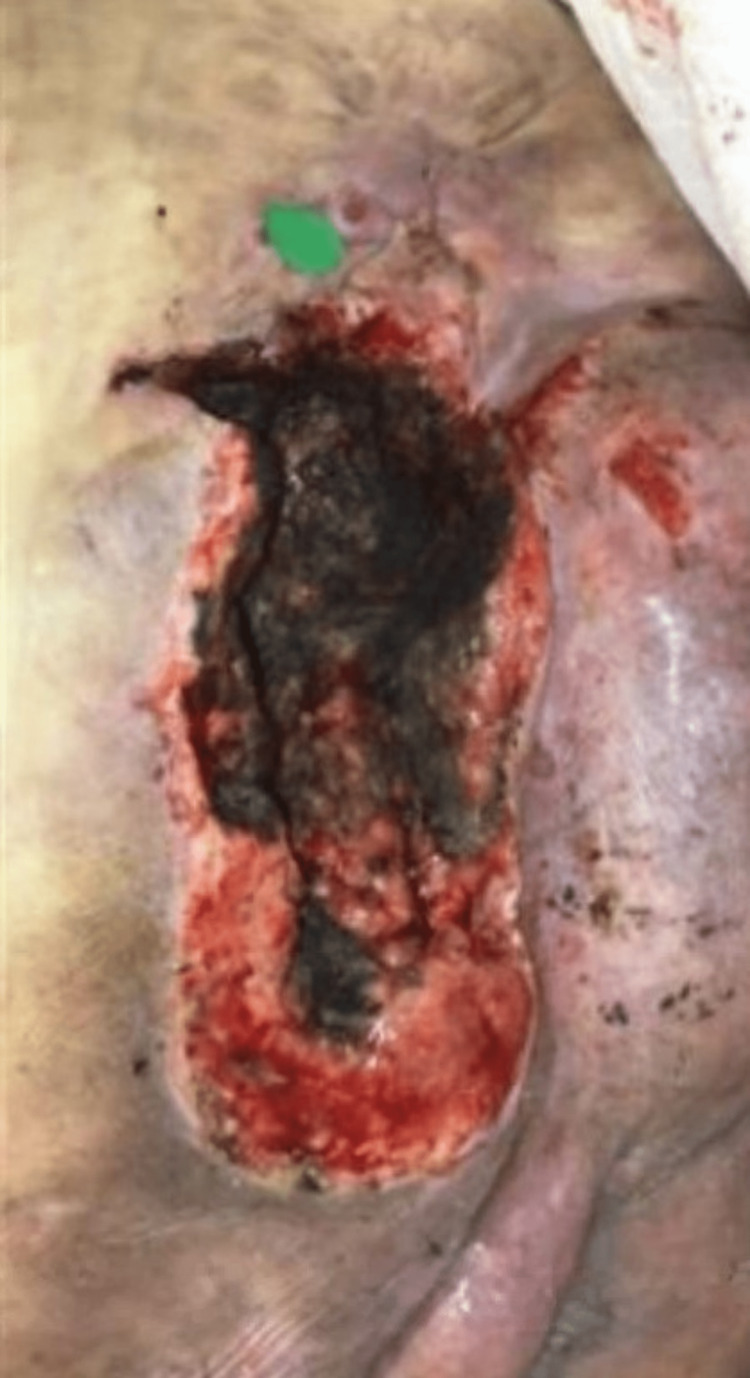
Unstageable left buttock pressure injury before surgery (18.26 x 8.22 x 7 cm). The depth of the ulcer is obscured by a black eschar within the wound bed.

A 59-year-old Caucasian male with a history of spina bifida, uncontrolled type 2 DM, and anemia presented for surgical consultation in the intensive care unit (ICU). The patient had a nonhealing sacral pressure ulcer on the left buttock for one year that had progressively worsened. He had a history of a right foot amputation, an amputation of three toes on the left foot, three sacral ulcer debridements at the bedside, and extensive wound care management. These interventions, however, failed to prevent wound advancement. The patient’s body mass index was 57.6 kg/m^2^. His medical history included supermorbid obesity, anemia, type II DM, end-stage chronic renal failure on dialysis, and osteomyelitis of the remaining right calcaneus. A review of the patient’s systems revealed pain in the lower back and gluteal region, loss of tactile sensation and motor function in the lower extremities, and multiple superficial lower extremity lacerations. The patient was afebrile. Physical examination revealed morbid obesity and immobility. Skin examination showed multiple lesions, including an 18.26 × 8.22 × 7 cm unstageable left buttock pressure injury. The base and floor of the ulcer were obscured by black eschar within the wound bed, and the margins were irregular and difficult to assess due to the presence of necrotic tissue (Figure 1).

**Figure 2:**
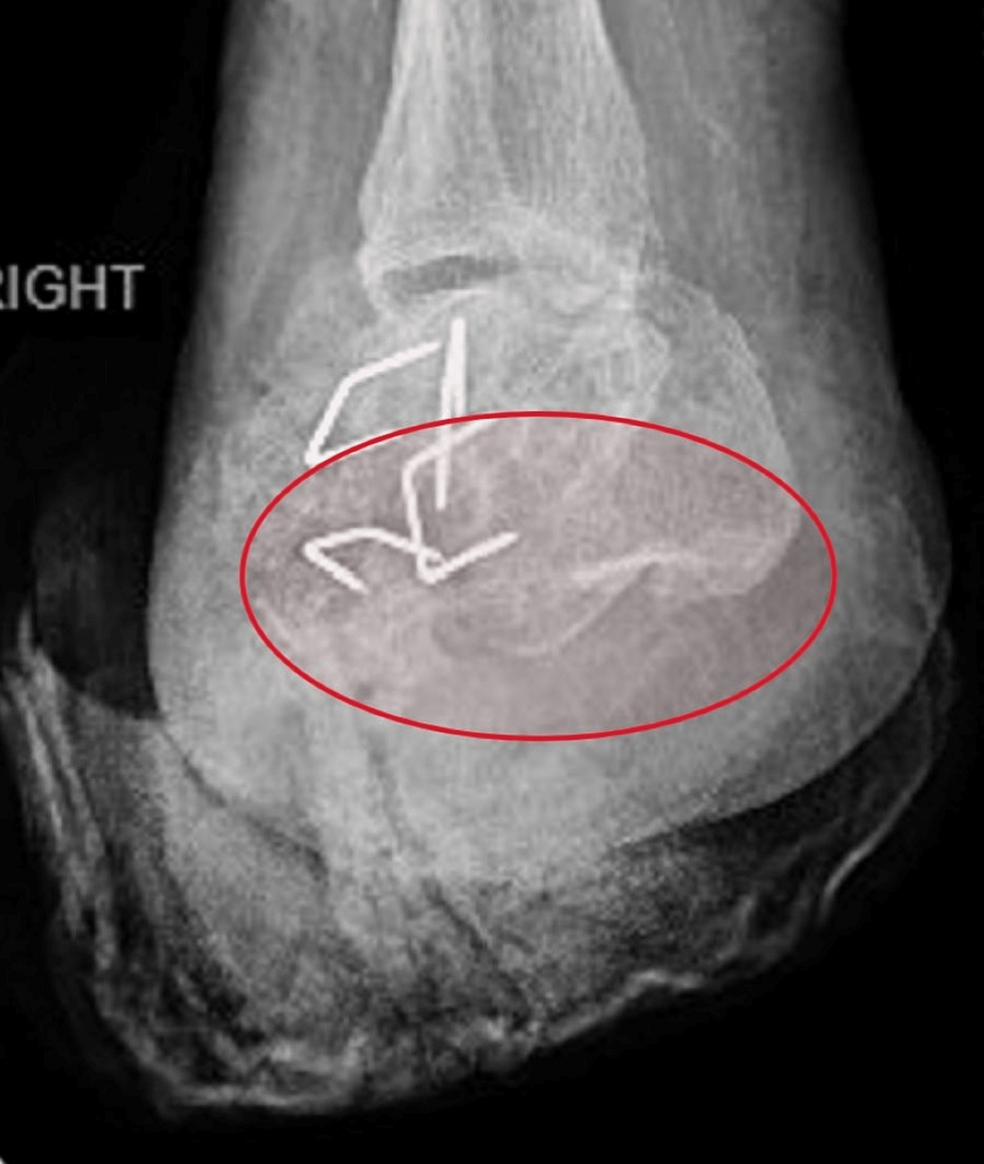
X-ray of the right foot and ankle showing postamputation changes of the hindfoot. The abnormal density of the remaining calcaneum and talus with erosive changes suggested the simultaneous presence of osteomyelitis.

Further examination revealed a right foot medial deep tissue injury with the depth of the ulcer obscured by a black eschar within the wound bed, a right lateral foot ulcer with full-thickness skin loss, a right foot plantar ulcer with full-thickness skin loss, and a well-healed right leg stump surgical incision. Relevant laboratory abnormalities included an elevated white blood cell count of 12,000/mL (normal range, 4,000-11,000/mL), elevated fasting serum glucose level of 184 mg/dL (normal range, 70-100 mg/dL), elevated C-reactive protein level of 10 mg/dL (normal value, <0.3 mg/dL), and an elevated erythrocyte sedimentation rate of 66 mm/h (normal range, 0-15 mm/h in men). Radiographs showed abnormal density of the remaining calcaneus and talus with erosive changes (Figure 2).

**Figure 3:**
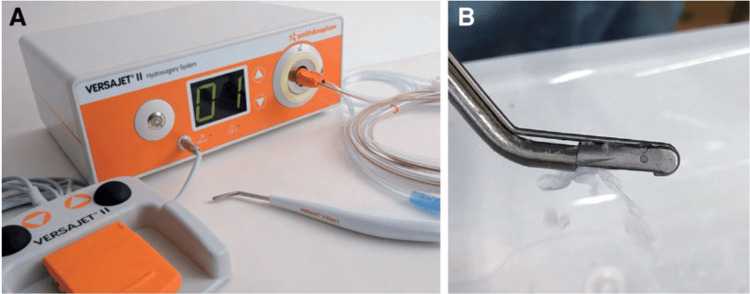
Hydrosurgery system (VERSAJET™). A) Body of the device. B) Handpiece for irrigation and simultaneous aspiration [11].

Histopathological analysis of the biopsy taken from the right foot confirmed the presence of osteomyelitis. Despite intravenous (IV) antibiotic treatment, healing of the sacral ulcer remained poor, and the patient experienced chronic pain. After obtaining proper informed consent from the patient, we conducted surgical intervention in the operating room (OR) involving debridement of the skin and soft tissue using high-power water via the VERSAJET™ Hydrosurgery System (Smith & Nephew, London, UK) (Figures 3A and B) [10].

**Figure 4:**
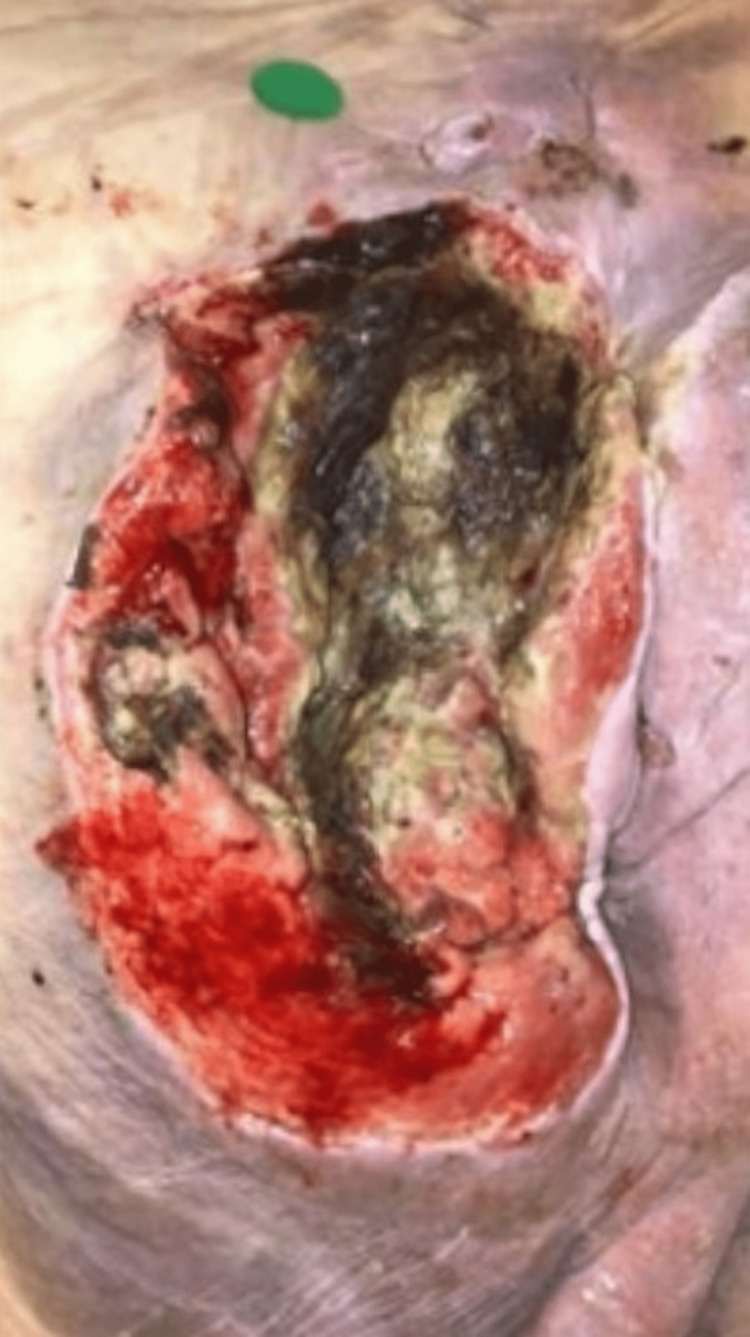
Stage four nonhealing left buttock pressure injury after surgery (18.54 x 11.98 x 7 cm). Necrotic skin and subcutaneous tissue extend below the gluteal muscles and into the sacral bone.

During debridement, ulcerated and necrotic skin and subcutaneous tissue extending below the gluteal muscles and into the sacral bone were observed. The wound was determined to be an 18.26 × 8.22 × 7 cm stage-four pressure ulcer (Figure 4).

A sacral bone biopsy was performed with a strong suspicion of osteomyelitis based on the clinical picture and laboratory results [5]. Postoperatively, the sacral bone biopsy confirmed osteomyelitis; deep cultures of bone results were positive for methicillin-resistant *Staphylococcus aureus*. One week postoperatively, the patient was returned to the OR for loop colostomy placement; the decision was made based on the assessment that the wound would not heal effectively due to its proximity to the rectum and the inevitable contamination with stool. Intraoperatively, the surgeon encountered challenges in determining whether all necrotic bone and tissue had been successfully removed, thereby indicating a probable need for repeat debridement. As a result, the patient was returned to the OR two times postoperatively for further debridement. After the final debridement, the patient's wound began to progressively heal.

## Discussion

This case study presents a complex scenario involving a patient with spina bifida, diabetes, obesity, and osteomyelitis. Our analysis investigates the intricate interplay of these multifactorial conditions, drawing parallels with existing literature while uncovering unique insights that contribute to our understanding of effective management strategies.

A review of the available literature suggests that osteomyelitis is relatively uncommon in patients with stage 4 sacral pressure ulcers. In Kenya, a previous study reported that 50% of patients with osteomyelitis had spina bifida [12]; however, these patients were primarily affected by foot osteomyelitis. In another case series by Dudareva et al., 67.2% of patients had osteomyelitis associated with pressure ulcers [13]. Furthermore, 64% of these patients were affected by central nervous system injury or pathology causing limitation in mobility. Among these patients, nine had diabetes and three were obese [13]. Similar to the case in our study, these studies suggest that patients with multiple comorbidities, including spina bifida, diabetes, and obesity, are at an increased risk for osteomyelitis.

The coexistence of spina bifida, diabetes, obesity, and osteomyelitis in our patient underscores the heightened vulnerability of individuals with such comorbidities to serious complications. Our findings align with the findings of prior studies that have highlighted the increased risk of osteomyelitis development in patients with neuropathic disorders, such as spina bifida, and diabetes-related foot ulcers [12-14]. Moreover, obesity has been recognized as a contributing factor to delayed wound healing and increased infection rates, further exacerbating the challenges faced by our patient [8].

A key takeaway from our case report is the pivotal role of a multidisciplinary approach in managing such complex medical cases. The successful outcome in our patient can be attributed to the collaborative efforts of general surgeons, endocrinologists, wound care specialists, and infectious disease experts. This echoes the recommendations of previous studies that emphasize the importance of integrated care in optimizing outcomes for individuals with complex health profiles [13].

While the findings of our case study align with existing literature in several aspects, we also reveal a unique challenge: the optimal timing of surgical intervention. Although early surgical debridement and antibiotic therapy have been conventionally recommended, our patient’s obesity presented logistical complexities that required meticulous preoperative planning. This observation prompts a nuanced consideration of patient-specific factors when determining the timing of surgical interventions, particularly in obese individuals [15]. Moreover, the atypical presentation of osteomyelitis in our patient highlights the need for heightened clinical suspicion. The absence of classical signs and symptoms in individuals with neuropathy emphasizes the importance of regular screenings and early diagnostic interventions to prevent advanced disease stages [1,6,16,17].

Among the differential diagnoses considered in our patient, we included sacral bone avascular necrosis (AVN). Radiographs typically do not show AVN in its early stages, as a negative radiograph does not rule out this diagnosis. AVN was considered a possibility because of the patient’s pain and gradual onset and progression of the wound. However, it was considered less likely because of the absence of joint involvement and steroid use [18]. Additionally, metastatic bone disease was considered as part of the differential diagnosis because of the patient’s pain and loss of motor function in the lower extremities. Metastatic bone disease was not considered a likely diagnosis owing to the absence of neoplasms. Alternative options were limited as different treatments failed to prevent the expansion of central wound necrosis. Repeated debridement without placement of a colostomy bag would have also been another option; however, we decided against this approach because the wound was extensive and located next to the rectum, which increased the risk of contamination and repeated wound infection.

In patients with myelomeningocele in Slovenia, Spazzapan et al. used colostomy placement in 5.2% of patients to manage bowel incontinence [16]. This study confirmed the idea that colostomy placement can help prevent sacral ulcer worsening by diverting stool away from the area, minimizing the risk of further skin irritation and breakdown as well as infection. Extended antibiotic monotherapy with vancomycin was another alternative option; however, we dismissed it as well because the disease was extensive, and antibiotics would poorly penetrate the necrotic bone [19].

In the literature, it has been observed that conservative treatment in the presence of skin ulceration is not a viable option and may facilitate bone infection [17,20]. According to previous research, there is limited evidence regarding the efficacy of antibacterial therapy in osteomyelitis without extensive surgical debridement and culture-based long-term parenteral antimicrobial therapy [1,20]. One study in the literature described a large cohort study of patients with sacral osteomyelitis who received one of three treatments: antibiotic therapy alone, both antibiotics and surgical debridement, or a myocutaneous flap. Those treated with a combined medical and surgical approach had a significantly decreased likelihood of being rehospitalized in the following year [20]. These findings comply with the conclusion that antimicrobial therapy alone without surgical debridement is less likely to lead to recovery in these patients.

This case report contributes to the existing knowledge by providing insight into the complex interplay between spina bifida, diabetes, obesity, and osteomyelitis. It highlights the significance of interdisciplinary collaboration and underscores the importance of tailoring interventions to individual patient characteristics. By contextualizing our findings within the broader framework of existing literature, we contribute to a more comprehensive understanding of managing complex cases and pave the way for more tailored therapeutic approaches in the future.

## Conclusions

Spina bifida, obesity, and DM can individually and collectively increase the risk of osteomyelitis through various interconnected mechanisms, including impaired immune function, poor circulation, neuropathy, reduced sensation, mechanical stress on bones, and impaired wound healing. The combination of these mechanisms creates a complex interplay of factors that significantly increases the risk of osteomyelitis. Proper management and preventive measures, including regular medical monitoring, weight management, blood sugar control, and diligent wound care, are crucial in reducing the risk of osteomyelitis and its potential complications. In the presented case, repeated debridement of the patient’s stage four sacral pressure ulcer improved the viability of the diseased tissue; however, a loop colostomy was performed to prevent necrotic tissue progression and recurrence. Therefore, future bone removal and sacral reconstruction should be considered. The presented findings underscore the importance of ongoing research and innovation in medical practices to further improve the quality of life for patients facing such intricate medical conditions.
